# Long-term polarization of alveolar macrophages to a profibrotic phenotype after inhalation exposure to multi-wall carbon nanotubes

**DOI:** 10.1371/journal.pone.0205702

**Published:** 2018-10-29

**Authors:** Kunihiro Otsuka, Koichi Yamada, Yuhji Taquahashi, Rieko Arakaki, Aya Ushio, Masako Saito, Akiko Yamada, Takaaki Tsunematsu, Yasusei Kudo, Jun Kanno, Naozumi Ishimaru

**Affiliations:** 1 Department of Oral Molecular Pathology, Tokushima University Graduate School of Biomedical Sciences, Tokushima, Japan; 2 Division of Cellular and Molecular Toxicology, Biological Safety Research Center, National Institute of Health Sciences, Kanagawa, Japan; 3 Department of Immunology and Parasitology, Tokushima University Graduate School of Biomedical Sciences, Tokushima, Japan; 4 Department of Pathology and Laboratory Medicine, Tokushima University Graduate School of Biomedical Sciences, Tokushima, Japan; 5 Japan Bioassay Research Center, Japan Organization of Occupational Health and Safety, Kanagawa, Japan; Helmholtz Zentrum Munchen Deutsches Forschungszentrum fur Umwelt und Gesundheit, GERMANY

## Abstract

**Background:**

Nanomaterials are widely used in various fields. Although the toxicity of carbon nanotubes (CNTs) in pulmonary tissues has been demonstrated, the toxicological effect of CNTs on the immune system in the lung remains unclear.

**Methods and finding:**

In this study, exposure to Taquann-treated multi-walled CNTs (T-CNTs) was performed using aerosols generated in an inhalation chamber. At 12 months after T-CNT exposure, alveolar inflammation with macrophage accumulation and hypertrophy of the alveolar walls were observed. In addition, fibrotic lesions were enhanced by T-CNT exposure. The macrophages in the bronchoalveolar lavage fluid of T-CNT-exposed mice were not largely shifted to any particular population, and were a mixed phenotype with M1 and M2 polarization. Moreover, the alveolar macrophages of T-CNT-exposed mice produced matrix metalloprotinase-12.

**Conclusions:**

These results suggest that T-CNT exposure promoted chronic inflammation and fibrotic lesion formation in profibrotic macrophages for prolonged periods.

## Introduction

Nanomaterials are manufactured chemical substances that are widely used in a variety of fields and exhibit novel characteristics, such as increased strength, chemical reactivity, and conductivity compared with the same materials without nanoscale features [1, 2]. Nanomaterials developed using nanotechnology have numerous potential applications in the fields of engineering, electronics, physics, chemistry, industry, biosciences, and medicine [3–5]. By contrast, changes to the environment by human activities, such as air pollution due to nanomaterial production, can adversely affect human health by the induction of various diseases [6–8]. However, the relationship between the specific features and pathogenesis of nanomaterials remains unclear.

Nanomaterials are foreign substances for the human body, which induce an immune response to clear foreign particles and protect health [9–11]. Many reports have demonstrated the toxicity of carbon nanotubes (CNTs) in the respiratory organs, especially the lungs [12–15]. The risk to human health has also been shown by the development of pulmonary inflammation and fibrosis in mice following inhalation of CNTs [16–19]. Alveolar macrophages play a central role in the engulfment and phagocytosis of CNTs in the lung [20, 21]. Alveolar macrophages no longer function after phagocytosis of CNTs, and activated macrophages cause injury to the alveolar epithelial cells via enhanced production of reactive oxygen species [22]. In addition, it has been reported that alveolar macrophages undergo cell death after engulfing CNTs, and the functional failure makes it difficult for the lungs to clear CNTs [23]. Moreover, the risk of pulmonary fibrosis is increased by long-term exposure of CNTs in mice [24]. Also, alveolar macrophages continue to accumulate in the lungs at 12 months after exposure to CNTs [24]. However, the phenotypic change and the function of alveolar macrophages in mice exposed to CNTs remain unclear.

A report demonstrated that pulmonary exposure to CNTs exacerbated inflammatory lesions in the lungs of mice infected with bacteria [25]. Because of the decrease in the cell number and the functional failure of alveolar macrophages, the clearance of bacteria in the lung becomes impaired [25]. Pharyngeal aspiration and intratracheal spray methods have been widely used in studies of CNT inhalation [19, 26]. However, changes in the particle size and/or shape of CNTs affect the nature and extent of toxicity in lung tissues [12, 27]. Taquahashi et al. recently reported a new method, named the “Taquann method,” and an apparatus to improve toxicological experiments using multiwall CNTs (MWCNTs) [28]. This method and the chamber with a direct injection system are expected to render inhalation toxicity studies of MWCNTs more relevant [28].

In this study, phenotypic changes and features of alveolar macrophages were analyzed in mice following the long-term exposure to Taquann-treated MWCNTs (T-CNTs) using a direct injection system in which well-dispersed aerosol is generated in an inhalation chamber. The findings of this research will be useful to further elucidate the relationship between alveolar macrophages and toxicity of nanomaterials.

## Materials and methods

### Ethics

This study was conducted according to the Fundamental Guidelines for Proper conduct of Animal Experiment and related Activities in Academic Research Institutions under the jurisdiction of the Ministry of Education, Culture, Sports, Science and Technology of Japanese Government. The protocol was approved by the Committee on the Animal Experiments of the University of Tokushima and Biological Safety Research Center, National Institute of Health Sciences (Permit Number: T27-7 and 601). All experiment was performed under anesthesia, and all efforts were made to minimize suffering.

### Mice

The protocol of this animal study was approved by the institutional ethics committee and conducted in accordance with the Guidance for Animal Studies of the National Institute of Health Sciences. Thirty, 8-week-old female C57BL/6NCrSlc mice (SLC, Inc., Shizuoka, Japan) were exposed to Taquann-treated MWCNTs (T-CNTs). At 12 months after exposure, tissues were collected from the mice for analysis.

### Taquan-treated multi-walled CNTs (T-CNTs) and whole body inhalation exposure

MWCNTs (Mitsui MWNT-7) were donated by Mitsui & Co., Ltd. (Tokyo, Japan). The Mitsui MWNT-7 is a mixture of dispersed single fibers of various length and width, and their agglomerates and aggregates. In order to obtain aggregates/agglomerates-eliminated, highly-dispersed fibers, pristine MWCNTs were treated by the Taquann method as described previously [28]. In brief, the method involves two processes; liquid-phase fine filtration and critical point drying to avoid re-aggregation by surface tension. MWCNT was suspended in Tert-butyl alcohol, freeze-and-thawed, filtered by a vibrating 25 μm mesh Metallic Sieve (Seishin Enterprise Co., LTD., Tokyo, Japan) snap-frozen by liquid nitrogen, and vacuum-sublimated. The average fiber length of T-CNT was identical to the pristine MWNT-7 (7.1 ± 6.0 μm in T-CNT, 7.1 ± 5.7 μm in pristine MWNT-7). Mice were exposed to the T-CNT aerosol 2hr per day in a week for 5 weeks (total 10 hr) by Taquann Direct-injection Whole Body inhalation System (version 2.0, manufactured by Sibata Scientific technology LTD., Saitama, Japan) [28]. The originally designed direct injection system is able to be generated well-dispersed aerosol in an inhalation chamber. Measured amount of dispersed T-CNTs were preloaded inside the cartridges and then compressed air were injected into the cartridges and blew out the T-CNTs through four small outlets of the cartridge into the subchamber, in which main flow air from mass flowmeter mixes in. The air with the aerosol goes down the connection pipe to the main chamber. Actual average mass concentrations of aerosol were 0, 1.42 and 3.12 mg/m^3^ in control, low dose and high dose, respectively.

### Histological analysis

All organs were removed from T-CNT-exposed mice, fixed with 10% phosphate-buffered formaldehyde (pH 7.2), and prepared for histological examination. Sections were stained with hematoxylin and eosin (H&E). Connective tissues of lung sections were detected by Azan staining. The area of the connective tissue was measured using Adobe Photoshop CS6 (Adobe Systems Incorporated, San Jose, CA, USA).

### Flow cytometric analysis

The bronchoalveolar lavage fluid (BALF) was collected with 1 ml of phosphate-buffered saline containing 2% fetal calf serum using an 18 G × 2" luer adapter as a tracheal cannula. BALF cells were collected by centrifugation at 400 g for 5min. Cells from BALF, spleen, and lymph nodes (LNs) were stained with antibodies against CD45.2, CD11b, F4/80, CCR2 (CD192), and CD206 conjugated to fluorescein isothiocyanate (FITC), phycoerythin (PE), allophycocyanin (APC), Per-Cy5.5, PE-Cy7, or APC–Cy7 (eBioscience, San Diego, CA). A FACScanto flow cytometer (BD Biosciences, Franklin Lakes, NJ) was used to identify the cell populations. Data were analyzed using FlowJo FACS Analysis software (Tree Star Inc., Ashland, OR).

### Scanning electron microscope (SEM)

Lung lobes were collected and treated with lysis solution composed of 5% potassium hydroxide, 0.1% sodium dodecyl sulfate, 0.1% ethylene-N,N,N’,N’-tetraacetic acid disodium salt dehydrate, and 2% ascorbic acid in ultra-pure water, dissolved at 80°C, and centrifuged at 20,000 g for 1 h at 25°C. The pellet containing T-CNT was recovered. In order to remove debris covering the fibers, 1.8 ml of 70% ethanol was added to the tube and incubated at 80°C for 30 min, and centrifuged at 20,000 g for 1 h at 25°C. 100 μl of 1% TritonX-100 was added to the pellet and dispersed by pipetting. 1 μl of the suspension was placed on an inorganic aluminum oxide membrane filter and filtrated on a funnel shape glass filter. The filer was dried at room temperature and osmium coated for SEM. SEM (VE-98, Keyence Co., LTD., Osaka, Japan) was used for detection of the ultrastructure of the samples.

### Confocal microscopic analysis

Free-floating fluoroimmunohistochemistry of the lung was performed with 60 μm-thick sections floating in solution in a 48-well plate. The sections were fixed with 4% paraformaldehyde phosphate buffer, permeabilized with 1% Triton, blocked with 10% goat serum (DAKO, Carpinteria, CA), and then stained with a rabbit anti- MMP-12 antibody (Abcam plc, Cambridge, UK) and FITC-conjugated rat anti-F4/80 antibody (eBioscience). After washing three times with 0.2% Triton, the sections were stained with Alexa 568 goat anti-rabbit immunoglobulin (Ig)G and Alexa 488 goat anti-FITC-IgG (Invitrogen Corporation, Carlsbad, CA). Nuclear DNA was stained with 4′,6-diamdino-2-phenylindole dihydrochloride (DAPI) (Invitrogen Corporation). Sections were observed using a PASCAL confocal laser-scanning microscope (LSM: Carl Zeiss, Jena, Germany) at 400× magnification. LSM image browser version 3.5 (Carl Zeiss) was used for image acquisition. The number of positive cells per square millimeter was calculated.

### Immunohistochemistry

For the immunohistochemical (IHC) analysis of lung tissues, paraffin-embedded sections were deparaffinized and subsequently applied to heat-induced antigen retrieval in HistoVT One (Nacalai Tesque). The sections were incubated with rabbit anti collagen IV antibody (Abcam). Protein binding was detected with a Vectastain elite ABC kit (Vector Laboratories Ltd, Peterborough, UK) and 3,3’-diaminobenzidine, tetrahydrochloride (DAB) as a substrate, and counterstained with hematoxylin.

### Quantitative reverse transcription-polymerase chain reaction (RT-PCR)

Total RNA was extracted from lung tissues using Isogen reagent (Wako Pure Chemical Industries, Ltd., Osaka, Japan) and subsequently reverse-transcribed into cDNA. Expression levels of mRNAs encoding MCP-1, iNOS, CD192, Arginase-1, Retnla, MGL-1, MGL-2, CHl3L1, IL-1β, IFN-γ, TNF-α, IL-12, IL-10, IL-13, IL-5, TGF-β 1, Col1A2, Col3A, ColIV, MMP-2, MMP-9, MMP-12, TIMP-2, TIMP-3, and β-actin were determined using a PTC-200 DNA Engine Cycler (Bio-Rad Laboratories, Hercules, CA) with SYBR Premix Ex Taq reagent (Takara Bio, Shiga, Japan). The primer sequences used were as follows: MCP-1, forward, 5′-CTGGATCGGAACCAAATGAG-3′, and reverse, 5′-TGAGGTGGTTGTGGAAAAGG-3′; iNOS: forward, 5′-CTGCAGCACTTGGATCAGGAACCTG-3′ and reverse, 5′- GGGAGTAGCCTGTGTGCACCTGGAA-3′; CD192, forward 5'-CCATGCAAGTTCAGCTGCCT-3', reverse 5'-TGCCGTGGATGAACTGAGG-3'; Arginase-1, forward, 5′-CAGAAGAATGGAAGAGTCAG-3′, and reverse, 5′-CAGATATGCAGGGAGTCACC-3′; Retnla: forward, 5′-TCCCAGTGAATACTGATGAGA-3′, and reverse, 5′-CCACTCTGGATCTCCCAAGA-3′; MGL-1: forward, 5′-AACCAATAGCAGCTGCCTTCATGC-3′, reverse, 5′-TGCAACAGCTGAGGAAGGACTTGA-3′; MGL-2: forward, 5′-GCATGAAGGCAGCTGCTATTGGTT-3′, reverse, 5′-TAGGCCCATCCAGCTAAGCACATT-3′; CHl3L1: forward, 5′-GATGGCCTCAACCTGGACTG-3′, reverse, 5′-CGTCAATGATTCCTGCTCCTG-3′; IL-1β: forward, 5′- ATGGCAACTGTTCCTGAACTCAACT-3′, and reverse, 5′- CAGGACAGGTATAGATTCTTTCCTTT-3′; IFN-γ: forward, 5′-AGCGGCTGACTGAACTCAGATTGTAG-3′, and reverse, 5′-GTCACAGTTTTCAGCTGTATAGGG-3′; TNF-α: forward, 5′-CCTCCTGGCCAACGGCATG-3′ and reverse, 5′-GCAGGGGCTCTTGACGGCAG-3′; IL-12: forward, 5′-TGGGAGTACCCTGACTCCTG-3′ and reverse, 5′-AGGAACGCACCTTTCTGGTT-3′; IL-10: forward, 5′-GCTCTTACTGACTGGCATGAG-3′, and reverse, 5′-CGCAGCTCTAGGAGCATGTG-3′; IL-13: forward, 5′-AGACCAGACTCCCCTGTGCA-3′ and reverse, 5′-TGGGTCCTGTAGATGGCATTG-3′; IL-5: forward, 5′-CGCTCACCGAGCTCTGTTG-3′ and reverse, 5′-CCAATGCATAGCTGGTGATTTTT-3′; TGF-β1: forward, 5′-GACCGCAACAACGCCATCTAT-3′, and reverse, 5′-GGCGTATCAGTGGGGGTCAG-3′; Col1A2: forward, 5′-CCAAGGGTAACAGTGGTGAA-3′ and reverse, 5′-CCATCACTGCCCCGAGCACC-3′; Col3A: forward, 5′-AACGGAGCTCCTGGCCCCAT-3′ and reverse, 5′-CCATCACTGCCCCGAGCACC-3′; Col IV: forward, 5′-ATGCCCTTTCTCTTCTGCAA-3′ and reverse, 5′-GAAGGAATAGCCGATCCACA-3′; MMP-2: forward, 5′-GACATACATCTTTGCAGGAGACAAG-3′ and reverse, 5′-TCTGCGATGAGCTTAGGGAAA-3′; MMP-9: forward, 5′-GAAGGCAAACCCTGTGTGTT-3′ and reverse, 5′-AGAGTACTGCTTGCCCAGGA-3′; MMP-12: forward, 5′-TGGTATTCAAGGAGATGCACATTT-3′ and reverse, 5′-GGTTTGTGCCTTGAAAACTTTTAGT-3′; TIMP-2: forward, 5′-CGTTTTGCAATGCAGACGTA-3′ and reverse, 5′-GATGGGGTTGCCATAGATGT-3′; TIMP-3: forward, 5′-CACGGAAGCCTCTGAAAGTC-3′ and reverse, 5′-TCCCACCTCTCCACAAAGTT-3′; β-actin, forward, 5′-GTGGGCCGCTCTAGGCACCA-3′, and reverse, 5′-CGGTTGGCCTTAGGGTTCAGGGGG-3′.

### Statistical analysis

Differences between individual groups were determined using one-way ANOVA. *p* < 0.05 was considered statistically significant. Power calculations were performed before the beginning of the experiments to determine the sample size for experiments using animals.

## Results

### Histological findings of lung tissues from T-CNT-exposed mice

**Fig 1:**
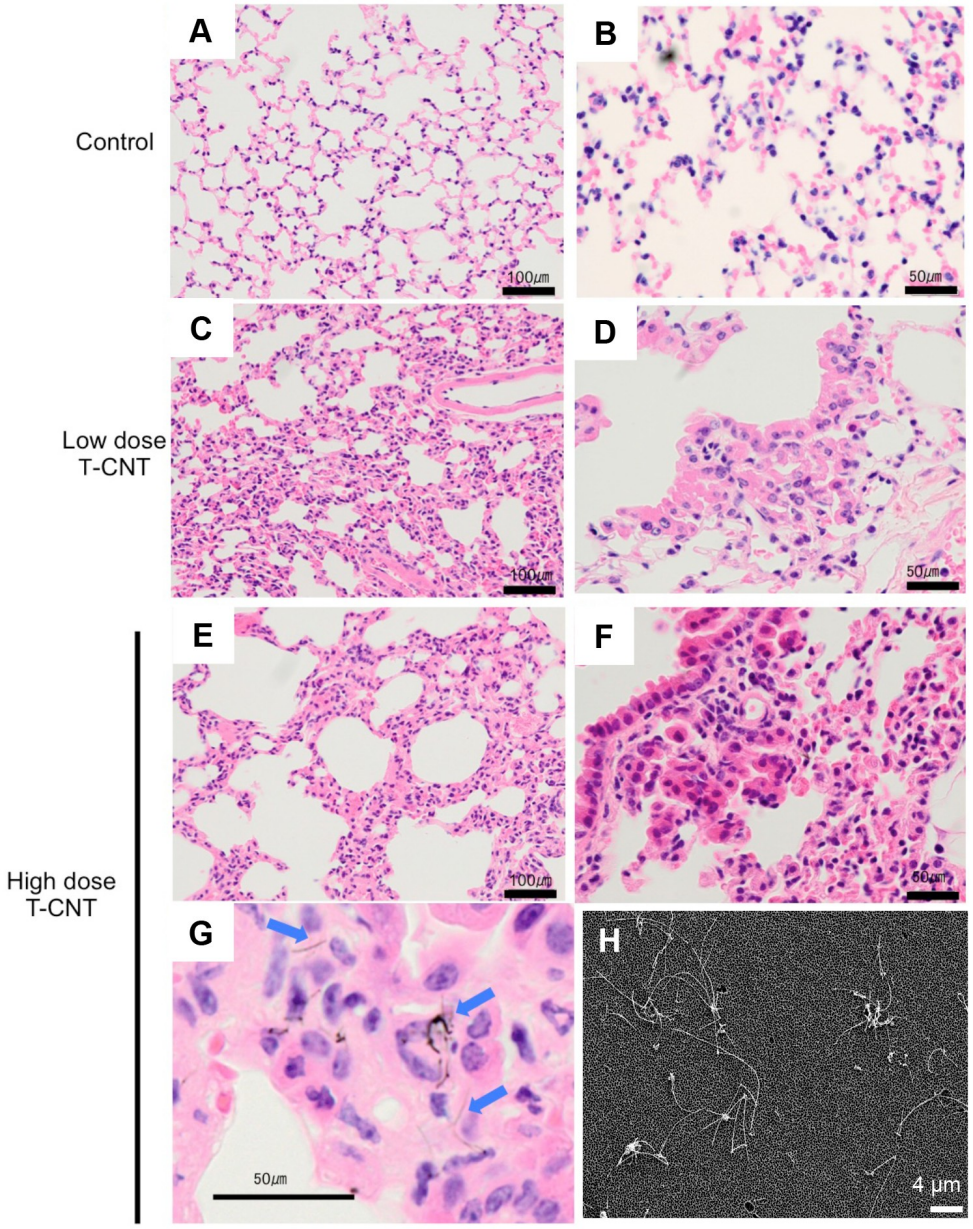
Pathology of lung tissues in T-CNT-exposed mice. Lung tissues at 12 months after T-CNT exposure were collected and tissue sections were stained with H&E. (A, B) Controls, (C, D) Low dose T-CNT, (E-G) High dose T-CNT. Change to the alveolar wall is shown (A-F). Macrophage accumulation in the alveolar space is shown (D-G). Photos are representative of each group. Scale bar: 100 μm (A, C, E), 50 μm (C, D, F, G). Arrow heads indicate T-CNT fibers (G). (H) SEM of the sediment of the dissolved lung tissue of a mouse exposed to T-CNT (high dose). Photos is representative of high dose group. Scale bar: 4 μm.

Normal female C57BL/6 mice were exposed to Taquann-treated multi-walled CNT (T-CNT) for 2 hr. a day in a week for 5 weeks using a whole body inhalation system as described previously [28]. Average mass concentrations of aerosol were 0, 1.42 and 3.12 mg/m^3^ in control, low dose and high dose, respectively. At 12 months after the last exposure, all mice were analyzed. Histological analysis of lungs tissues collected from the T-CNT-exposed (low & high dose T-CNT) mice showed thickening of the alveolar wall, as compared with control mice (Fig 1A, 1C and 1E). In addition, there was accumulation of monocytes in the alveolar space of T-CNT-exposed and control mice (Fig 1B, 1D and 1F). The pulmonary structure of mice exposed to high dose T-CNT was unclear in some area due to hypertrophy of the alveolar wall and monocyte accumulation in the alveolar space (Fig 1C~1F). Single fibers of T-CNT were diffusely detected within the alveolar wall and phagocytes (Fig 1G). Aggregation of T-CNT fibers was hardly observed in the lung of T-CNT-exposed mice. In addition, T-CNT fibers were recovered from the lung tissue of T-CNT-exposed (high dose) mice, and were detected by a scanning electron microscope (SEM). Dispersed single fibers were observed (Fig 1H). These findings demonstrate that phagocytosis of T-CNT by alveolar macrophages continues for long periods after exposure to T-CNT, suggesting that the alveolar immune system may fail to clear CNTs for prolonged periods after exposure.

### Fibrotic change in lung tissues by T-CNT exposure

**Fig 2:**
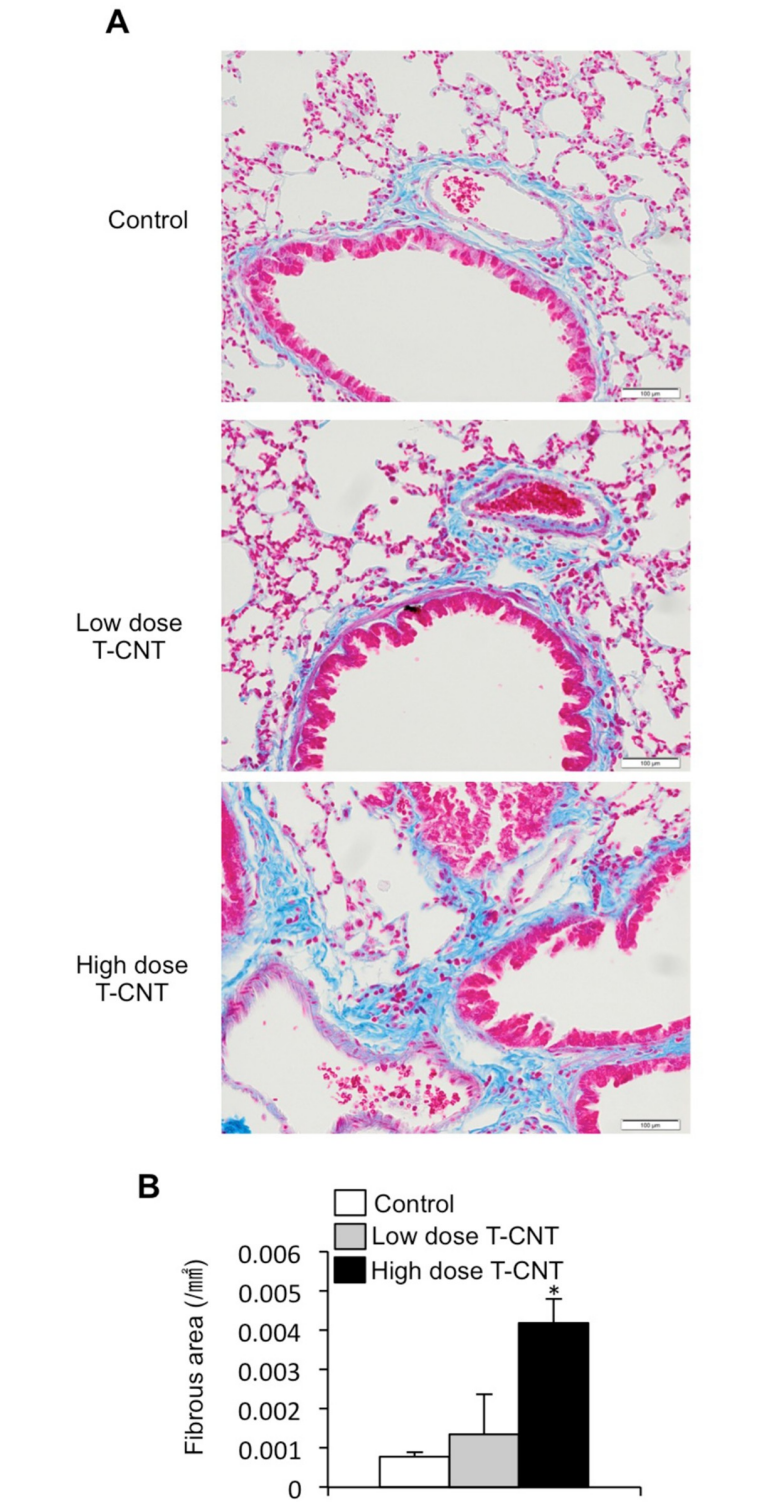
Fibrotic lesions in the lung of T-CNT-exposed mice. (A) Connective tissues in the lung of T-CNT-exposed mice were detected by Azan staining. Photos are representative of each group. Scale bar: 100 μm. (B) The area (mm^2^) of Azan-stained connective tissue was measured. Data are presented as the average ± standard deviation (SD) of five mice of each group. *p < 0.05, vs controls.

In addition to inflammatory lesions of the alveolar space and wall, marked interstitial fibrosis was observed in the lung tissues of T-CNT-exposed mice (Fig 2A). Histological analysis by Azan staining showed that proliferation of collagen fiber was promoted by T-CNT exposure (Fig 2A). There was a significant increase in fibrosis around the bronchi and blood vessels within the interstitial area in the lungs of T-CNT-exposed mice, as compared with control mice (Fig 2B). These findings suggest that besides prolonging alveolar inflammation, T-CNT exposure also induces chronic inflammation in the interstitial area for long periods after exposure.

### Alveolar macrophages in T-CNT-exposed mice

**Fig 3:**
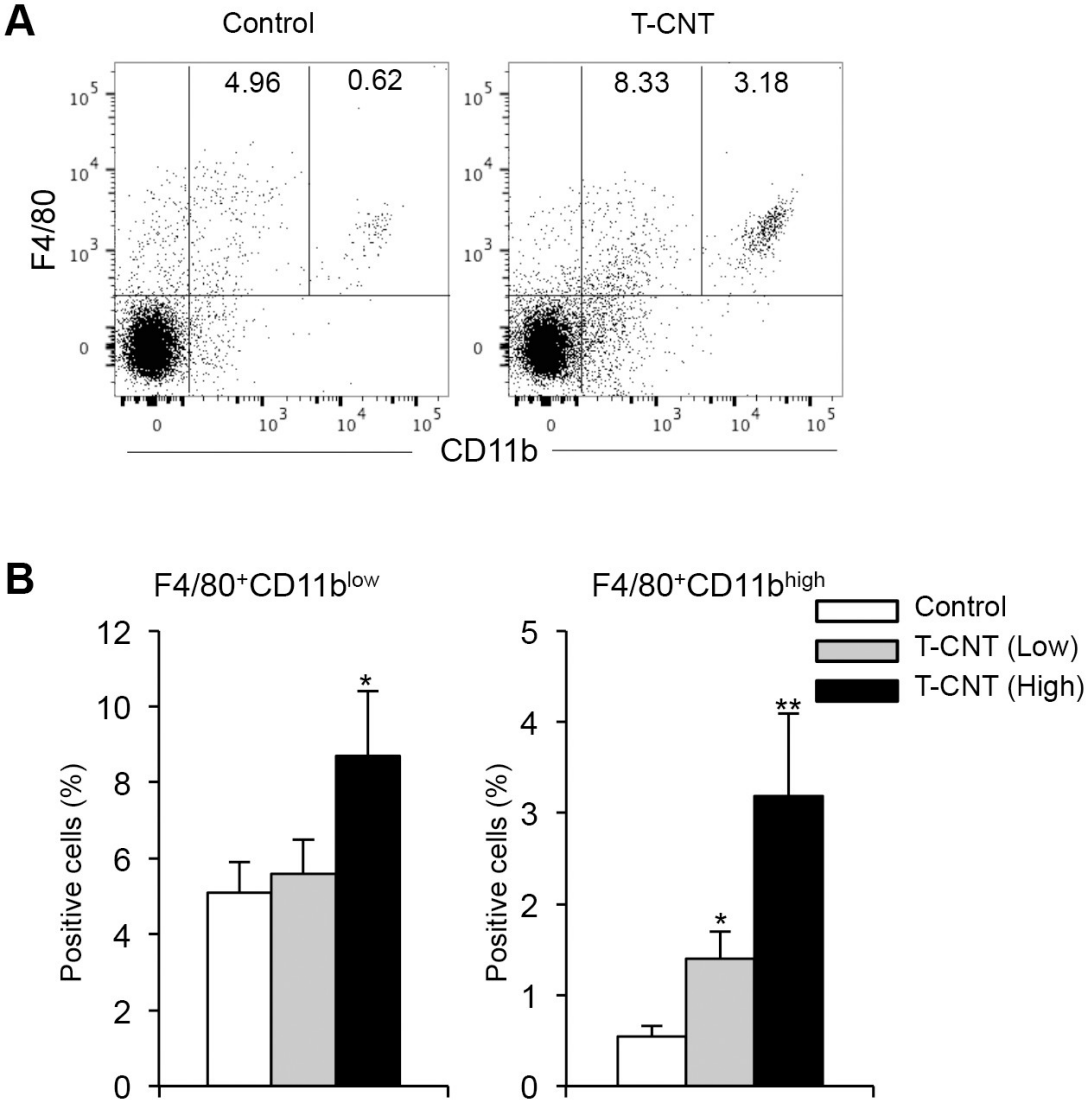
Alveolar macrophages in T-CNT-expose mice. (A) CD11b and F4/80 expression of mononuclear cells in BALF from control and T-CNT-exposed mice were detected by flow cytometric analysis. The results are representative of each group. F4/80^+^CD11b^low^ and F4/80^+^CD11b^high^ populations are shown. (B) The proportion of F4/80^+^CD11b^low^ and F4/80^+^CD11b^high^ macrophage in BALF from control and T-CNT-exposed mice were detected by flow cytometric analysis. Data are presented as the average ± SD of five mice of each group. *p < 0.05, **p < 0.005, vs controls.

Next, flow cytometric analysis of alveolar macrophages using mononuclear cells in bronchoalveolar lavage fluid (BALF) collected from control and T-CNT-exposed mice was performed. The surface phenotype of almost alveolar macrophages in normal mice is considered to be F4/80^+^CD11b^low^. By exposure to T-CNT (high dose), both F4/80^+^CD11b^low^ and F4/80^+^CD11b^high^ populations were significantly increased, as compared with those of control mice (Fig 3A and 3B). These findings demonstrate that long-term exposure to T-CNT induces accumulation of alveolar macrophages.

### Phenotype of alveolar macrophage in T-CNT-exposed mice

**Fig 4:**
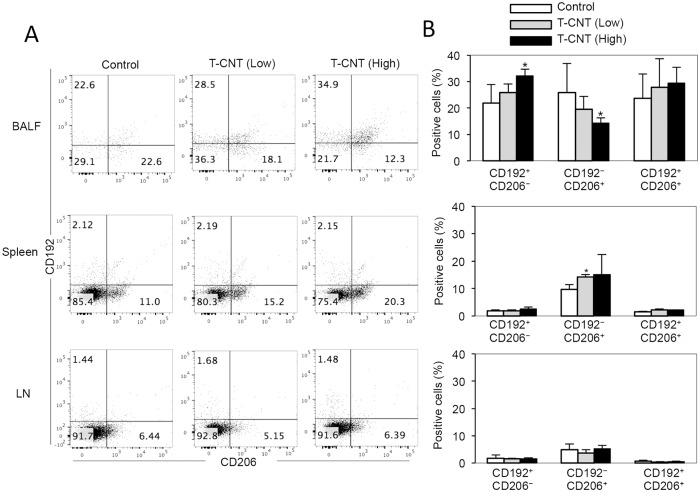
M1-like and M2-like macrophage phenotype of alveolar macrophages in T-CNT-exposed mice. (A) CD192 (M1) and CD206 (M2) expression of F4/80^+^CD11b^+^ macrophages in BALF, spleen, and LNs was detected by flow cytometric analysis. The results are representative of each group. (B) The proportion of CD192^+^CD206^−^ M1-like macrophages, CD192^−^CD206^+^ M2-like macrophages, and CD192^+^CD206^+^ macrophages in BALF, spleen, and LNs was measured flow cytometric analysis. Data are presented as the average ± SD of five mice of each group. *p < 0.05, vs controls.

Furthermore, the phenotype of alveolar macrophages in BALF was analyzed by detection of CD192, a marker of M1 macrophages, and CD206, a marker of M2 macrophages, among F4/80^+^CD11b^+^ macrophages. Compared with control mice, the proportion of CD192^+^CD206^−^ M1-like macrophages was significantly increased in BALF collected from mice exposed to a high dose T-CNTs (Fig 4A and 4B). By contrast, the proportion of CD192^−^CD206^+^ M2-like macrophages was significantly decreased by exposure to a high dose T-CNTs, as compared with that of control mice (Fig 4A and 4B). However, populations of CD192^+^CD206^+^ cells were similar between control T-CNT-exposed mice (Fig 4A and 4B). Although M1-like macrophages increased in T-CNT (high dose)-exposed mice, there was no clear shift to M1 or M2 macrophage differentiation of BALF cells in the mice exposed to T-CNT. These results demonstrate that T-CNT exposure sustains alveolar inflammation and doesn’t largely change M1 and M2 polarization of macrophage phenotype, showing a mixed type including M1, M2, and the other phenotype as a whole.

To determine the systemic effects of T-CNT exposure on the immune system, phenotypic changes of F4/80^+^CD11b^+^ macrophages in the spleen and lymph nodes were analyzed using CD192 and CD206 as markers of M1 and M2 macrophages. The proportion of CD192^−^CD206^+^ M2-like macrophages in the spleen of mice exposed to low doses of T-CNTs was significantly higher than that of control mice (Fig 4A and 4B). There were no changes in the proportion of the other populations in the spleen and LNs (Fig 4A and 4B). Thus, this finding suggests that pulmonary exposure to T-CNTs may influence differentiation or migration of macrophages in the spleen.

### Expression of macrophage-associated genes of lung in T-CNT-exposed mice

**Fig 5:**
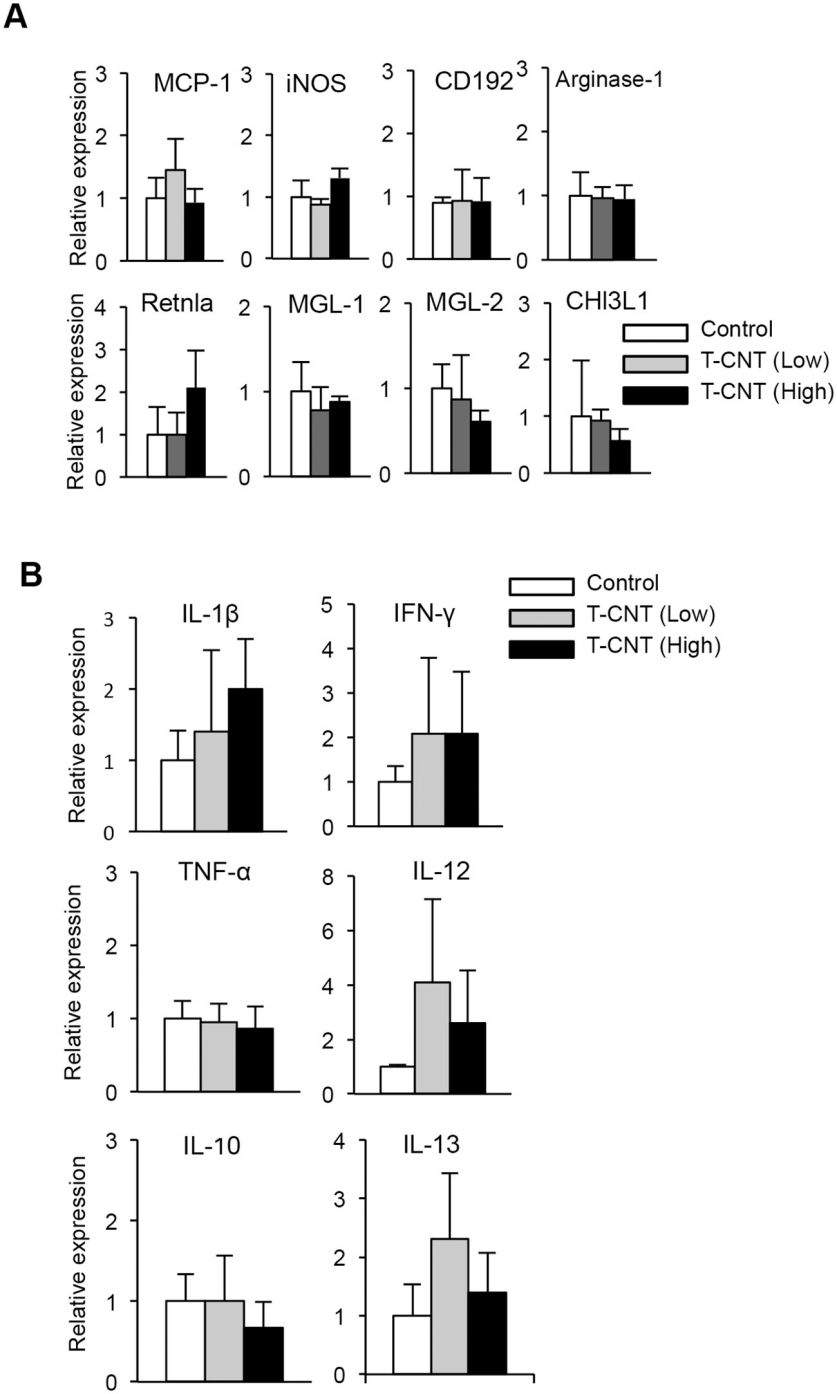
Macrophage-related gene expression in lung tissues of T-CNT-exposed mice. mRNA expression of macrophage-related genes of lung tissues in control and T-CNT-exposed mice was analyzed by real time RT-PCR. (A) Levels of mRNA encoding MCP-1, iNOS, CD192, Arginase-1, Retnla, MGL-1, MGL-2, and CHl3L1. Data are presented as the average of relative expression to control ± SD of five mice of each group. (B) Levels of mRNA encoding IL-1β, IFN-γ, TNF-α, IL-12, IL-10, and IL-13. Data are presented as the average of relative expression to control ± SD of five mice of each group.

To further clarify the characteristics of alveolar macrophages in T-CNT-exposed mice, mRNA expression levels of M1 and M2 macrophage-associated genes of the lung tissues were analyzed by real time RT-PCR. The expression levels of monocyte chemotactic protein-1 (MCP-1), inducible nitric oxide synthase (iNOS), and CD192 mRNA, as M1 macrophage-associated genes, were not changed by T-CNT exposure (Fig 5A). Also, there was no change in the expression level of Arginase-1, resistin like alpha (Retnla), macrophage galactose-type lectin-1 (MGL-1), MGL-2, and chitinase-3-like protein-1 (CHl3L1) mRNA, M2 macrophage-associated genes, between control and T-CNT-exposed mice (Fig 5A). In addition, mRNA expression of cytokines, including interleukin (IL)-1β, interferon-γ (IFN-γ), tumor necrosis factor-α (TNF-α), and IL-12, which are derived from M1 macrophages, were analyzed by real time-RT-PCR using lung tissues. There were no significant differences in cytokine mRNA expression levels between control and T-CNT-exposed mice (Fig 5B). Moreover, there were no changes in mRNA expression levels of IL-10 and IL-13 in the lung tissues between control and T-CNT-exposed mice (Fig 5B). These findings support that T-CNT exposure doesn’t affect any particular polarization including M1 and M2 phenotype in alveolar macrophages.

### Profibrotic phenotype of alveolar macrophages in T-CNT-exposed mice

**Fig 6:**
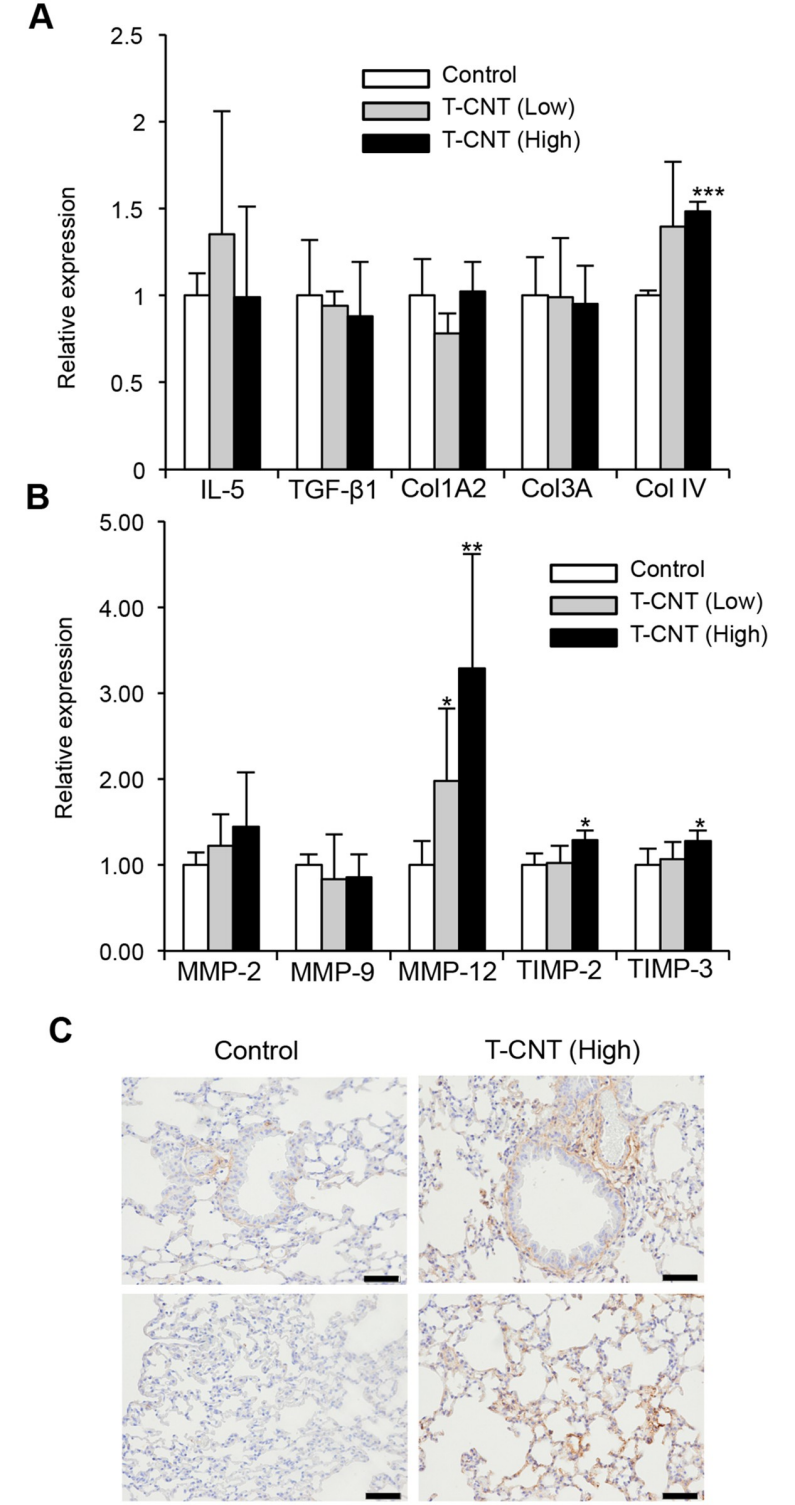
Gene expression showing profibrotic phenotype and collagen type IV expression in the lung tissues of T-CNT-exposed mice. (A, B) Levels of mRNAs encoding IL-5, TGF-β1, Col1A2, Col3A, ColIV, MMP-2, MMP-9, MMP-12, TIMP-2, and TIMP-3 were detected by real time RT-PCR. Data are presented as the average of relative expression to control ± SD of five mice of each group. *p < 0.05, **p < 0.005, vs controls. (C) Protein expression of collagen type IV was detected by immunohistochemical analysis. Photos are representative of each group. Scale bar: 100 μm.

Next, the unique characteristics of alveolar macrophages following T-CNT exposure, were determined in T-CNT-exposed mice with enhanced fibrosis (Fig 2). Pulmonary fibrosis occurs by excess deposition of collagen-rich extracellular matrix through a variety of molecules produced by immune cells including monocytes/macrophages. Among them, collagen IV (Col IV), matrix metalloproteinase-12 (MMP-12), tissue inhibitors of metalloproteinase-2 (TIMP-2), and TIMP-3 mRNA expression levels were significantly increased in the lung tissues of mice exposed to high doses of T-CNTs (Fig 6A and 6B). In particular, expression levels of MMP-12 mRNA was markedly and dose-dependently increased by T-CNT exposure (Fig 6B). By contrast, there were no significant changes in the IL-5, transforming growth factor-β1 (TGFβ-1), ColA2, and Col3A mRNA expressions between control and T-CNT-exposed mice (Fig 6A). In addition, immunohistochemical analysis showed that collagen type IV expression in the stromal area around bronchus and vessel, and alveolar wall was enhanced by T-CNT exposure (Fig 6C). Therefore, the alveolar macrophages in T-CNT-exposed mice may account for the profibrotic phenotype.

### MMP-12 expression of alveolar macrophages in T-CNT-exposed mice

**Fig 7:**
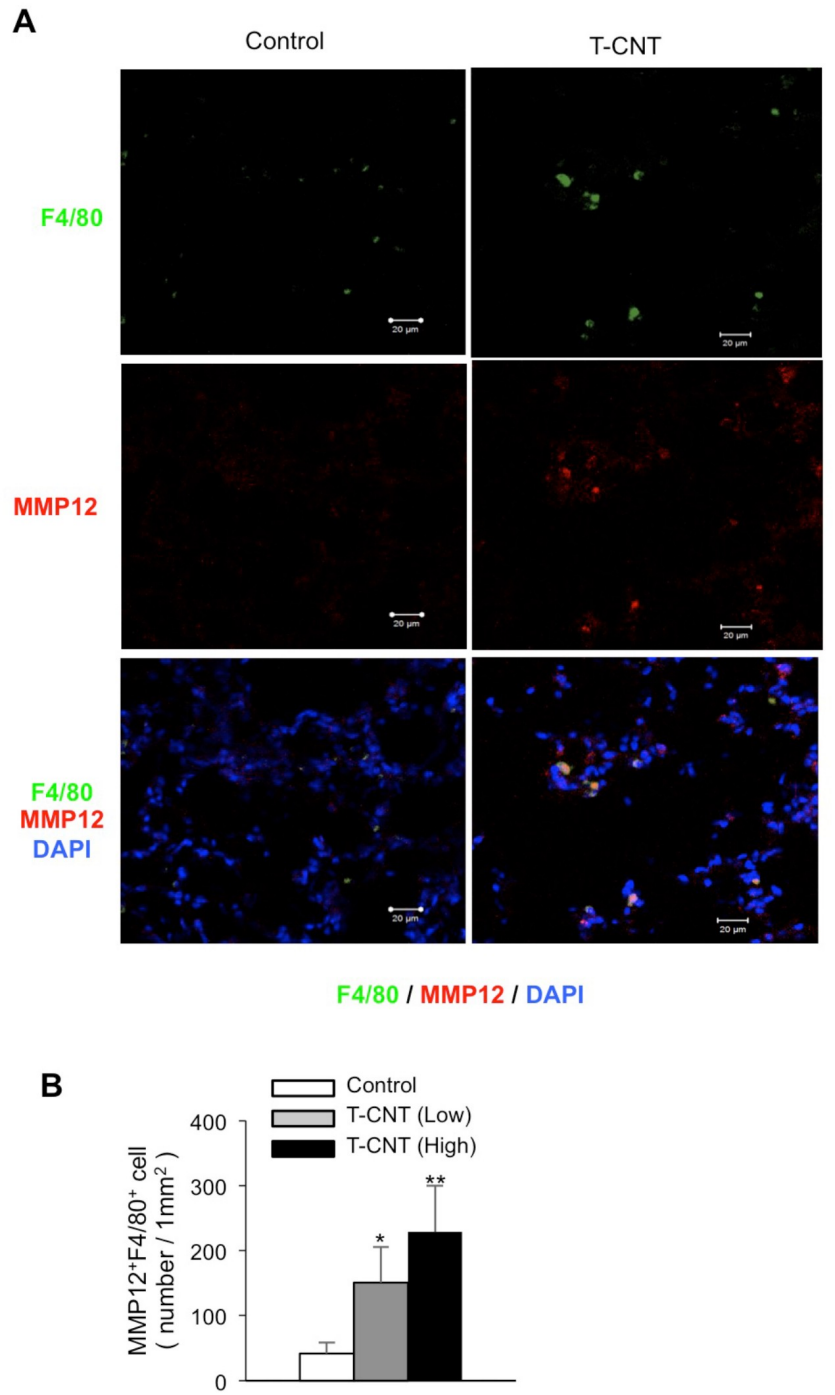
Detection of MMP-12-expressing alveolar macrophages in T-CNT-exposed mice. (A) MMP-12 and F4/80 expression using frozen lung tissues of control and high-dose T-CNT-exposed mice were analyzed by confocal microscopy. Nuclei were stained with DAPI. Photos are representative of five mice of each group. (B) Number (mm^2^) of F4/80^+^MMP-12^+^ alveolar macrophages was counted. Data are presented as the average of relative expression to control ± SD of five mice of each group. *p < 0.05, **p < 0.005, vs controls.

To confirm the presence of MMP-12 protein in alveolar macrophages in T-CNT-exposed mice, confocal microscopic analysis was performed using frozen lung tissues. A significant portion of F4/80^+^ alveolar macrophages in T-CNT-exposed mice expressed MMP-12, but only rarely in control mice (Fig 7A). In addition, the number of F4/80^+^MMP-12^+^ macrophages in lung tissues of T-CNT-exposed mice was significantly increased, as compared with that of control mice, in a dose-dependent manner (Fig 7B). Therefore, alveolar macrophages may display the profibrotic phenotype by T-CNT exposure.

## Discussion

Exposure to nanomaterials is known to induce various diseases, including the formation of pulmonary lesions [12, 21]. Many studies of CNTs have demonstrated the inability of alveolar macrophages to engulf CNT fibers, which promote the formation of pulmonary lesions in which reactive oxygen species derived from activated alveolar macrophages cause injury to alveolar epithelial cells ultimately resulting in to cell death [13, 14, 22, 23]. However, it remains unclear whether that aggregation/agglomerate of CNT fibers influences the activity of alveolar macrophages and formation of pulmonary lesions. In the present study, MWCNT fibers were treated by the Taquann method in order to remove aggregate/agglomerate and enrich the well-dispersed single fibers in a dry state without any dispersants. Furthermore, the mice were exposed to T-CNT using a whole body inhalation system which enables to be inhaled well-disperse MWCNT fibers to mice. In fact, we observed single MWCNTs fibers in alveolar regions, therefore the direct effect of CNT exposure on alveolar immune cells was evaluated in this study. Single fibers were diffusely observed in the lung of T-CNT-exposed mice, and the size of granulomatous lesions consisting of macrophage aggregation were relatively smaller than that of untreated MWCNT-exposed mice in the previous report [19]. The direct effect of CNT exposure on alveolar immune cells was evaluated by the application of a T-CNT injection system [28]. In actual human, exposure to nanomaterials results in alveolar lesions by single fibers. Because, the aggregate/agglomerate will form sediments quickly in the ambient air and will be effectively filtered out in human upper respiratory tracts. By contrast, in practical inhalation studies of experimental animals, the animal chamber air is rigorously agitated in order to ensure the homogeneity of aerosol. When given as a mixture, the likelihood of aggregates and agglomerates reaching the animal nose is high, and they would disturb inhalation of single fibers and induce bronchitis/bronchiolitis with granuloma. Thus, we concluded that it is essential to prepare a dispersed single fiber aerosol without aggregates and agglomerates, and further without changes in size and shape of single fiber components.

Macrophages play key roles in various immune responses during inflammation in a variety of tissues [29]. In addition to functions in innate immunity, such as antigen phagocytosis and cytokine production, antigen presentation by macrophages represents a link between innate and acquired immunity [30]. During the inflammatory processes, naïve monocytes differentiate into pro-inflammatory M1 and anti-inflammatory M2 macrophages [29, 30]. Macrophages originate from at least three sources, including the yolk sac, fetal liver, and bone marrow while alveolar macrophages are derived from the yolk sac and bone marrow [30]. Actually, various differentiated macrophages migrate and exist within the alveolar space in response to inflammatory stimuli. In this study, although the proportion of M1-like macrophages was significantly increased by high-dose T-CNT exposure, we concluded that long-term T-CNT exposure sustains alveolar inflammation and a mixed type macrophage differentiation including M1 and M2 phenotypes as a whole. In addition, there was no increase in expression levels of M1 and M2 macrophage-related genes in T-CNT-exposed mice. A previous report described that MWCNTs can induce macrophages into a mixed phenotype, part M1 and part M2 macrophage [31]. Therefore, the unique population of alveolar macrophages by T-CNT exposure might promote chronic pulmonary inflammation. On the other hand, the concentration dependency on the effect of T-CNT in alveolar immune response was partial in this study. It is possible that any threshold of functional ability by pulmonary macrophage may affect the effect on the pulmonary immune response in T-CNT-exposed mice. On the other hand, the proportion of CD11b^high^ macrophage and the expression of MMP12 were dependent on the concentration of T-CNT.

Macrophages are involved in various functions, including tissue repair, fibrosis formation, and angiogenesis [32]. Alveolar macrophages play a resolution-promoting role during the reversible phase of bleomycin-induced pulmonary fibrosis [33, 34]. In addition, macrophages contribute to both the induction and resolution phases of acute lung injury [35]. Thus, there has been much controversy regarding the role of monocytes and macrophages in the pathogenesis of pulmonary fibrosis. When an allergy model of house dust mite allergen was exposed to MWCNTs, the allergic response was prevented through suppression of IL-1β and pro-caspase-1 in alveolar macrophages [36]. In addition, MWCNT-induced airway fibrosis was enhanced by allergen challenge [36]. These results suggest that MWCNT-induced inflammasome regulated in an allergy inflammatory microenvironment could play an important role in increased airway fibrogenesis. A recent DNA microarray study demonstrated expression of a wide range of cytokine and chemokine genes in the lung tissues of mice at 1 year after MWCNT exposure [24].

Fibrotic lesion formation in the lung is caused by excessive deposition of interstitial collagens [37]. Maintenance of the extracellular matrix and tissue repair is controlled by MMPs [37]. Various MMPs and cytokines, including MMP-2, 3, 7, 8, 9, 12, 13, TIMPs, IL-5, and TGF-β1, play profibrotic roles in lung injury and inflammation [37, 38]. Among these molecules, MMP-12 is known as a macrophage metalloelastase that is produced by activated macrophages and contributes to fibrotic lesion formation in the lungs [39–41]. In this study, MMP-12 mRNA in the lung tissues of T-CNT-exposed mice, as compared with that of control mice, was markedly increased. Further, MMP-12 production by F4/80^+^ alveolar macrophages was confirmed in T-CNT-exposed mice. These findings suggest that MMP-12-producing alveolar macrophages are involved in the pathogenesis of the fibrotic lesions for long periods after T-CNT exposure.

## Conclusions

In conclusion, use of the newly established Taquann method and aerosol generation system showed that formation of chronic inflammatory lesions in mice continues for long periods after CNT exposure. The alveolar macrophages in T-CNT-exposed mice were sustained in the state of a M1/M2 mixed macrophage phenotype. In addition, the profibrotic character of alveolar macrophages was demonstrated in T-CNT-exposed mice. The findings of this research should prove helpful to further elucidate the toxicological effects of nanomaterials on the pulmonary immune system.

## Abbreviations

CNT: carbon nanotube
MMP-12: matrix metalloprotinase-12
T-CNTs: Taquann-treated CNT
